# Comprehensive Analysis of Outcomes and Complications in Free Tissue Transfer for Breast Reconstruction: A Retrospective Study From a Lower-Middle-Income Country (LMIC)

**DOI:** 10.7759/cureus.78613

**Published:** 2025-02-06

**Authors:** Syeda Afifa, Taha Ahmad, Muhammad Omar Afzal, Muhammad Hamza Naeem Jaan, Noor Fatima Chaudhry, Tooba Sabir, Talha Bin Nasir, Hassan Shahid, Yashfeen Ahmed

**Affiliations:** 1 Plastic and Reconstructive Surgery, Shaukat Khanum Memorial Cancer Hospital and Research Centre, Lahore, PAK; 2 General Surgery, Shaukat Khanum Memorial Cancer Hospital and Research Centre, Lahore, PAK; 3 Community Medicine, Al-Aleem Medical College, Lahore, PAK; 4 Pediatrics, Dow Medical College, Dow University of Health Sciences, Karachi, PAK; 5 Physiology, Lahore Medical and Dental College, Lahore, PAK; 6 Surgical Oncology, Shaukat Khanum Memorial Cancer Hospital and Research Centre, Lahore, PAK

**Keywords:** autologous reconstruction, breast reconstruction, free tissue transfer, microsurgery, surgical outcomes

## Abstract

Introduction

Autologous breast reconstruction using free tissue transfer is widely regarded as the gold standard for oncologic reconstruction due to its reliability and high patient satisfaction. However, it is associated with challenges such as prolonged operative time, extended recovery, and potential complications. This study aims to comprehensively evaluate early and late complications, their impact on length of stay, and readmissions in a retrospective study from a single institution.

Methods

A retrospective review of a maintained free flap database was conducted, identifying patients who underwent free tissue transfer for breast reconstruction at Shaukat Khanum Memorial Cancer Hospital and Research Centre from 2022 to 2024. Patient demographics, history of cancer, surgery performed, and complications (both intraoperative and postoperative) were analyzed. Complications were categorized into minor, immediate major, and delayed major, with rates calculated per patient. Statistical analysis was performed using the chi-square test, with significance set at p < 0.05.

Results

A total of 12 patients underwent 13 free tissue transfers, including deep inferior epigastric artery perforator flaps in four patients (30.77%), profunda artery perforator flaps in five patients (38.46%), anterolateral thigh flaps in three patients (23.08%), and superior gluteal artery perforator flaps in one patient (7.69%). The average operative time was seven hours and 16 minutes, with a mean hospital stay of 5.69 days. The overall flap success rate was 92.31%. Minor complications occurred in four patients (30.77%), while delayed major complications were observed in one (7.69%) patient. Complete flap loss was reported in one patient, linked to preexisting pulmonary comorbidities.

Conclusions

Free tissue transfer remains a reliable option for breast reconstruction, but patient comorbidities and intraoperative factors significantly influence outcomes. Improved preoperative counseling, close intraoperative monitoring, and prompt management of complications enhance patient satisfaction and overall success rates.

## Introduction

Breast cancer is one of the most commonly diagnosed malignancies worldwide, with mastectomy often being a necessary component of its treatment [1]. For many patients, breast reconstruction is an essential part of the healing process, addressing both physical and emotional recovery [2]. Among the various reconstructive options, autologous breast reconstruction using free tissue transfer has emerged as the preferred approach due to its ability to provide natural results, long-term durability, and high levels of patient satisfaction [3]. Techniques such as the deep inferior epigastric artery perforator (DIEP) flap, profunda artery perforator (PAP) flap, anterolateral thigh (ALT) flap, and superior gluteal artery perforator (SGAP) flap offer distinct advantages, including minimal donor-site morbidity and superior aesthetic outcomes [4,5].

Despite its benefits, free tissue transfer is a technically demanding procedure, requiring advanced microsurgical expertise [6]. It is associated with potential challenges, including prolonged operative times, extended recovery periods, and a range of complications [7]. The overall success of these procedures is primarily defined by flap survival, with reported rates exceeding 90%; however, complications - both minor and major - remain a significant concern [8]. These complications can profoundly affect patient outcomes, influencing recovery, satisfaction, and overall healthcare costs.

Although there is substantial literature on free tissue transfer for breast reconstruction, limited data exist regarding the differentiation between early and late complications and their specific impacts on postoperative outcomes, such as hospital stay and readmission rates [9-11]. Moreover, the role of patient-related factors and comorbidities in predicting complications warrants further investigation to optimize patient selection and perioperative management strategies [12].

This study aims to comprehensively evaluate the outcomes of free tissue transfer for breast reconstruction, focusing on the incidence and types of complications. By exploring their association with patient characteristics, comorbidities, and surgical variables, this research seeks to enhance understanding of the factors influencing surgical outcomes and inform strategies for improving patient care in this complex field.

## Materials and methods

Study design

We retrospectively reviewed our institutional free flap database to identify all oncologic breast reconstruction patients who underwent free tissue transfer at Shaukat Khanum Memorial Cancer Hospital and Research Centre from 2022 to 2024. The study was approved by the Institutional Review Board (approval number EX-03-01-25-05) and adhered to the Declaration of Helsinki.

Data collection

Data were extracted from hospital records, including preoperative histories, physical examinations, operative notes, anesthesia and nursing records, discharge summaries, outpatient notes, and laboratory reports. Additional queries were made in the hospital database to identify medical complications and readmissions related to reconstructive procedures.

Variables analyzed

Specific variables analyzed included baseline patient characteristics and comorbidities (age, BMI, hypertension, chronic obstructive pulmonary disease (COPD), hyperlipidemia, active smoking, coronary artery disease, and peripheral arterial disease); oncologic history (type of mastectomy, preoperative and postoperative chemotherapy, and prior irradiation); and reconstructive details (immediate versus delayed reconstruction, unilateral versus bilateral reconstruction, thrombotic events, flap type, and recipient vessel). Intraoperative complications (venous or arterial thrombosis) and postoperative surgical complications (any thrombosis, flap loss, delayed breast or donor-site wound complications, early infections during hospitalization, delayed outpatient infections, and seroma) were also reviewed [13].

Obesity was defined using the World Health Organization classification system: class I obesity (BMI of 30-34.9 kg/m²), class II obesity (BMI of 35.0-39.9 kg/m²), and class III obesity (BMI greater than 40 kg/m²) [14]. Surgical complications were categorized as minor (wound healing issues, seroma, infection, transfusion, partial flap loss, and fat necrosis), immediate major (thrombosis and complete flap loss), and delayed major (hernia or donor-site dehiscence requiring intervention) [13]. Delayed wound healing at the abdominal donor site or mastectomy flap was defined as skin necrosis or wound breakdown requiring topical care or dressing changes for more than three weeks [15]. Fat necrosis was defined as a palpable firmness greater than 1 cm in diameter observed during follow-up, unrelated to cancer recurrence [16]. Partial flap loss was defined as loss or atrophy of up to 50% of the flap, not requiring an immediate return to the operating room [17].

Statistical analysis

Data was analyzed using IBM SPSS Statistics for Windows, Version 20.0 (Released 2011; IBM Corp., Armonk, NY, USA) with statistical significance set at p < 0.05.

## Results

A total of 12 patients underwent 13 free tissue transfers, including one bilateral and 11 unilateral reconstructions. The average follow-up period was six months, with no reported mortalities. All complications were analyzed and reported as per-patient incidence. The types of flaps utilized are shown in Table 1, with the DIEP flap being used in four (30.77%) cases, PAP flap in five (38.46%), ALT flap in three (23.08%), and SGAP flap in one (7.69%).

**Table 1:** Types of flaps utilized ALT, anterolateral thigh; DIEP, deep inferior epigastric artery perforator; PAP, profunda artery perforator; SGAP, superior gluteal artery perforator

Flap type	Number of patients (n)	Percentage (%)
DIEP flap	4	30.77%
PAP flap	5	38.46%
ALT flap	3	23.08%
SGAP flap	1	7.69%

The mean duration of the operations was calculated to be seven hours and 16 minutes, while the mean length of hospital stay was 5.69 days. The overall flap success rate was 92.31%.

Surgical complications

Complete flap loss occurred in one patient, accounting for one (7.69%) of the cases, as shown in Table 2. There were no major immediate surgical complications in any patient. However, one (7.69%) patient experienced a major delayed surgical complication, which was linked to a preexisting pulmonary infection identified as a comorbidity. Minor surgical complications were observed in four (30.77%) patients. Of these, three (23.08%) patients required minor surgical interventions during the delayed postoperative period, necessitating a return to the operating room.

**Table 2:** Surgical complications

Complication type	Number of patients (n)	Percentage (%)
Complete flap loss	1	7.69%
Major immediate surgical complications	0	0%
Major delayed surgical complications	1	7.69%
Minor surgical complications	4	30.77%
Minor surgical interventions (delayed)	3	23.08%

Based on our statistical analysis using the chi-square test as shown in Table 3, no significant association was found between minor complications and patient comorbidities (chi-square value = 2.84, degrees of freedom = 3, p-value = 0.42). However, a significant correlation was observed between major complications and preexisting pulmonary conditions (chi-square value = 6.21, degrees of freedom = 2, p-value = 0.045), highlighting the increased risk in this subset of patients. Additionally, gluteal artery-based flaps demonstrated a higher failure rate compared to abdominally based flaps (chi-square value = 5.67, degrees of freedom = 1, p-value = 0.017).

**Table 3:** Comparison

Comparison	Chi-square value	Degrees of freedom	p-Value	Significance
Minor complications vs. patient comorbidities	2.84	3	0.42	Not significant
Major complications vs. preexisting pulmonary conditions	6.21	2	0.045	Significant
Gluteal artery-based flaps vs. abdominally based flaps	5.67	1	0.017	Significant

This data underscores the overall high success rate of the free flap procedures (92.31%), with a low rate of major complications. Additionally, the findings suggest that preexisting conditions, such as pulmonary infections, can contribute to delayed complications in oncologic breast reconstruction cases.

## Discussion

The data from our hospital and the work conducted on free tissue transfer for breast reconstruction provide a comprehensive overview of the associated outcomes. No correlation was identified between minor complications and patient comorbidities. However, one major complication was observed, which was linked to a preexisting pulmonary condition. Patients with COPD undergoing free tissue transfer require careful consideration and thorough preoperative discussions. The increased risk in such cases is likely attributable not solely to COPD itself but to the coexistence of other comorbid conditions in this patient population.

A critical finding of our study is the association of gluteal artery-based flaps with a higher rate of flap loss compared to abdominally based flaps [18]. Gluteal artery flaps, although described for breast reconstruction, are known to have higher rates of flap loss and greater technical challenges due to their shorter pedicle length and vessel size discrepancies [19]. Our findings support this approach, prompting us to consider gluteal artery-based flaps as secondary or tertiary choices for free autologous reconstructions. This approach is due to their higher complication rates and the need for intraoperative repositioning.

Another important finding is that patient comorbidities independently predispose patients to an increased risk of complications. Obesity, for instance, has been associated with higher rates of flap-related complications, including flap loss, hernia, seroma, and delayed wound healing [20]. Interestingly, none of the patients in our study fell into the morbid obesity category, highlighting the need for individualized risk assessments in this population.

Despite these insights, our study has several limitations. The primary limitation is the relatively small sample size, which restricts the generalizability of our findings. Additionally, as a study conducted in a lower-middle-income country (LMIC), financial constraints often prevent widespread access to complex reconstructive procedures such as free tissue transfer [21]. Many patients in our setting are unable to afford such extensive procedures, limiting the number of eligible candidates and potentially affecting the overall complication rates observed. Furthermore, the availability of microsurgical expertise and advanced postoperative monitoring may not be as extensive in LMICs as in high-income countries, which could influence surgical outcomes [22]. Larger, multicenter studies are needed to better evaluate these challenges and refine strategies to improve accessibility and outcomes for patients in resource-limited settings.

The success of free tissue transfer in breast reconstruction has been largely dependent on consistent microsurgical techniques, intraoperative collaboration and communication with the anesthesia team, meticulous clinical monitoring of the flap, and prompt identification and management of vascular complications [13]. A deeper understanding of the risk factors and complications has enabled us to improve preoperative counseling, fostering open discussions that promote patient autonomy and informed decision-making. This approach has not only enhanced the management of complications but has also led to greater patient satisfaction.

## Conclusions

Free tissue transfer for breast reconstruction is an effective technique with high success rates, although complications vary based on flap type and patient comorbidities. In LMICs, financial constraints and limited microsurgical expertise pose challenges. Improved accessibility, meticulous perioperative management, and further research with larger cohorts are essential to optimize outcomes and expand reconstructive options in resource-limited settings.
